# A preclinical study demonstrating the efficacy of nilotinib in inhibiting the growth of pediatric high-grade glioma

**DOI:** 10.1007/s11060-015-1744-y

**Published:** 2015-03-04

**Authors:** Karolyn Au, Sanjay K. Singh, Kelly Burrell, Nesrin Sabha, Cynthia Hawkins, Annie Huang, Gelareh Zadeh

**Affiliations:** 1 The Arthur and Sonia Labatt Brain Tumour Research Centre, Hospital for Sick Children, University of Toronto, Toronto, ON M5G 1L7 Canada; 2 Division of Neurosurgery, Toronto Western Hospital, 4-439 West Wing, 399 Bathurst Street, Toronto, ON M5T 2S8 Canada

**Keywords:** Pediatric glioma, Nilotinib, PDGFRα, AKT signaling, ERK1/2 signaling

## Abstract

Solid tumors arising from malignant transformation of glial cells are one of the leading causes of central nervous system tumor-related death in children. Recurrence in spite of rigorous surgical and chemoradiation therapies remains a major hurdle in management of these tumors. Here, we investigate the efficacy of the second-generation receptor tyrosine kinase inhibitor nilotinib as a therapeutic option for the management of pediatric gliomas. We have utilized two independent pediatric high-grade glioma cell lines with either high platelet-derived growth factor receptor alpha (PDGFRα) or high PDGFRβ expression in in vitro assays to investigate the specific downstream effects of nilotinib treatment. Using in vitro cell-based assays we show that nilotinib inhibits PDGF-BB-dependent activation of PDGFRα. We further show that nilotinib is able to decrease cell proliferation and anchorage-independent growth via suppression of AKT and ERK1/2 signaling pathways. Our results suggest that nilotinib may be effective for management of a PDGFRα-dependent group of pediatric gliomas.

**Electronic supplementary material:**

The online version of this article (doi:10.1007/s11060-015-1744-y) contains supplementary material, which is available to authorized users.

## Introduction

Malignant glial neoplasms comprise 8–10 % of primary pediatric central nervous system (CNS) tumors [1], and are the most common cause of solid tumor-related death in children [2]. These lesions are classified as grade III or IV by the World Health Organization (WHO), and histology is most commonly anaplastic astrocytoma (AA; grade III) and glioblastoma (GBM; grade IV) [3]. The cornerstones of therapy remain surgical resection and adjuvant chemoradiation, but tumor recurrence is inevitable and long-term response to treatment remains extremely poor. Development of novel therapies has been restricted by limited understanding of tumor biology and dependence upon preclinical models based on adult disease.

Genomic studies of pediatric high-grade gliomas (HGG) have demonstrated that the most frequent copy number aberration (CNA) is focal amplification of the platelet-derived growth factor receptor (*PDGFRA*) locus at chromosome 4q12, encoding the α isoform of platelet-derived growth factor receptor (PDGFRα) [4–7]. Several exome sequencing profiles of high-grade pediatric supratentorial and brainstem gliomas revealed a frequent, novel mutation of *H3F3A*, encoding histone variant H3.3, that is associated with poorer overall survival [8, 9]; among brainstem lesions, amplification of *PDGFRA* occurred exclusively in *H3F3A*-mutant tumors [10]. PDGFRα protein expression corresponds to gene amplification, and is also seen in some tumors that do not contain *PDGFRA* amplification [11]. Expression of PDGFR correlates with high-grade histology in pediatric gliomas [12], and associated activation of downstream Ras- and AKT-pathway signaling mediators has been correlated with poor survival [13, 14].

The second-generation receptor tyrosine kinase inhibitor nilotinib (Tasigna; Novartis Pharmaceuticals) binds its targets with similar affinity to the prototype molecule imatinib (Gleevec; Novartis Pharmaceuticals) [15–19]. Unlike imatinib, it is not dependent upon the OCT-1 transporter for cell influx [20], and although it may be subject to transporter-dependent efflux [21, 22], it is detectable within the brains of mice with an intact blood–brain barrier [23]. Currently in use for treating imatinib-resistant and newly diagnosed chronic myeloid leukemia, nilotinib has a well-established safety and toxicity profile [24, 25]. In this study, we examine the effects of nilotinib on pediatric GBM-derived cell lines, in order to understand the biochemical and biological impact of inhibiting PDGFR signaling and to evaluate its suitability as a therapeutic target.

## Materials and methods

### Cell lines and culture

Pediatric GBM cell lines SJ-G2 and SF-188 [26, 27] were a gift from Dr. Nada Jabado (McGill University, Montreal, Quebec), and normal human astrocytes (NHA) immortalized with E7 and hTERT were a gift from Dr. Russell Pieper (University of California, San Francisco, CA, USA). Adult GBM cell lines U-118, U-87 and U-251 were obtained from the American Type Culture Collection (ATCC). All cell lines were cultured as adherent monolayers in minimal media supplemented with 10 % fetal bovine serum (FBS), at 37 °C in 5 % CO2.

### Evaluation of exogenous ligand stimulation and inhibition

Methods and results are presented below for SJ-G2; parallel experiments carried out in SF-188 are presented in supplementary sections (see detailed methods in Supplement). Culture stimulation was performed using dimerized B-isoform of recombinant human platelet-derived growth factor (PDGF-BB; Cell Signaling Technology), which has affinity for both α and β isoforms of PDGFR. Cell cultures at 70–80 % confluence were incubated overnight in growth media containing 0.5 % FBS, then washed with warm phosphate-buffered saline (PBS), and exposed to fresh minimal media containing PDGF-BB at working concentration. Stimulation was terminated by placing cultures on ice and washing with cold PBS, then scraping and suspending in cell lysis buffer.

### Western immunoblot assay

Cell cultures were scraped and lysed on ice in 1X Cell Lysis Buffer (Cell Signaling) containing protease (Roche Diagnostics) and phosphatase inhibitors (Calbiochem). Protein was quantified using bicinchoninic acid (BCA) assay (Thermo Scientific), and lysate containing 25–40 μg protein was loaded onto 7–12.5 % SDS-PAGE gels for electrophoresis. A detailed list of antibodies used is presented in Supplementary methods.

### Cell viability, proliferation and colony formation assays

Cell viability was assessed by MTS assay using CellTiter 96^®^ Aqueous One Solution reagent (Promega) according to the manufacturer’s instructions. Direct count of viable cells using the trypan blue dye exclusion method was performed with the Vi-CELL Cell Viability Analyzer (Beckman Coulter). For cell proliferation assay, chemiluminescent Cell Proliferation ELISA (Roche) using 5-bromo-2′-deoxyuridine (BrdU) labeling was carried out according to the manufacturer’s instructions, using a 12-h labeling period. Clonogenic assay and soft-agar colony-forming assay were performed to evaluate in vitro growth potential [28]. Colonies were fixed in 10 % formalin, then stained with 0.05 % crystal violet. Each condition was carried out in triplicate, in three independent experiments. Detailed methods are described in Supplementary methods.

### Subcutaneous xenograft

SJ-G2 cells (1 × 10^6^) were injected into the right flank of 6–8-week-old NOD–SCID mice (Jackson Laboratory). On detection of palpable tumor, mice were given nilotinib or vehicle by oral gavage (10 mg/kg daily), and sacrificed upon signs of sickness or at the end of the 10-week study. Excised tumor dimensions were measured with calipers, and volume estimated by the formula 4π*abc*/3, where *a*, *b* and *c* represent tumor radius in three planes. Animal use protocols were approved by the animal care committed of the Hospital for Sick Children (Toronto, Canada) under AUP#2656.

## Results

### PDGFR protein expression in pediatric GBM cell lines

**Fig. 1:**
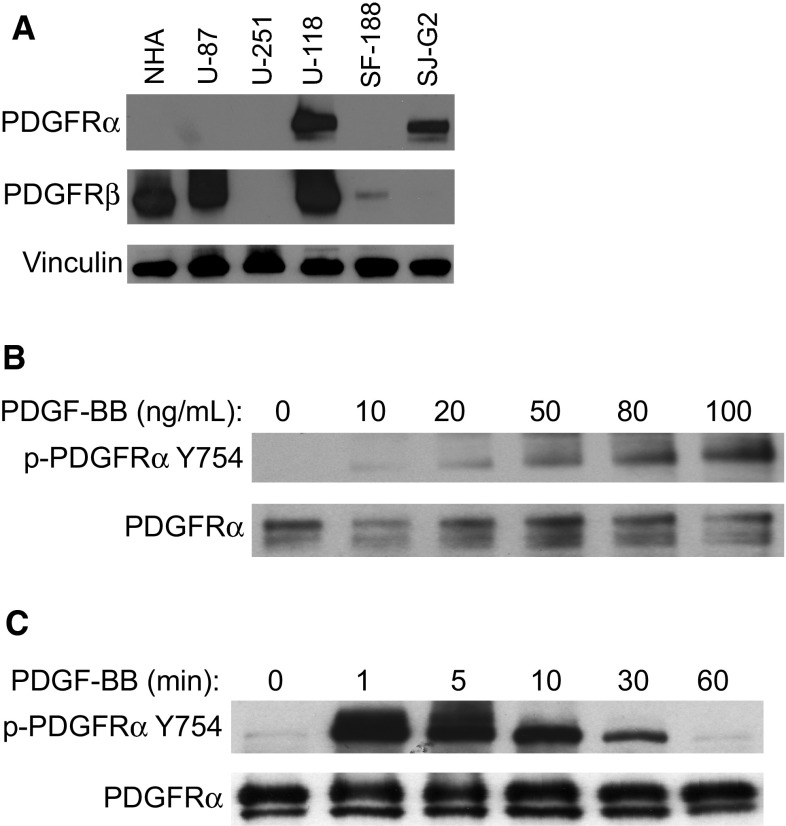
Expression and activation of PDGFRα. **a** Variable expression of PDGFRα and β is demonstrated in pediatric (SF-188, SJ-G2) and adult (U-87, U-251, U-118) glioblastoma cell-lines. **b** Western blot analysis of PDGFRα phosphorylation following exposure to exogenous PDGF-BB under varying conditions. Vinculin is loading control. SJ-G2 cells were exposed to 0–100 ng/mL PDGF-BB for 5 min. A concentration-dependent increase in PDGFRα phosphorylation was observed, to a plateau ≥50 ng/mL. **c** SJ-G2 cells were exposed to 100 ng/mL PDGF-BB for 0–60 min. Phosphorylation of PDGFRα occurred rapidly, with a peak observed following 1 min of ligand exposure

To assess relative expression levels of PDGFR proteins, the pediatric GBM cell lines SJ-G2 and SF-188 were compared to adult GBM cell lines and to non-transformed human astrocytes using immunoblotting. We found that NHA expressed PDGFRβ but not PDGFRα. SF-188 followed this pattern, by expressing minimal PDGFRβ and not PDGFRα. In contrast, SJ-G2 contained large amounts of PDGFRα, and far less PDGFRβ (Fig. 1a). Results for SJ-G2 are presented throughout, and parallel experiments carried out in SF-188 are presented in Supplementary Data and Figures. Neither pediatric GBM cell line was found to contain high-level amplification of PDGFRA (Supplementary Fig. 1S). In summary, pediatric GBM—like adult GBM—contains variable expression of the two PDGF receptors.

### Activation of PDGFRα is PDGF-BB-dependent

When grown in low-serum conditions, SJ-G2 cells contained minimal phosphorylated PDGFRα. Addition of exogenous PDGF-BB to growth media resulted in phosphorylation of PDGFRα. Phosphorylation increased with increasing ligand concentration to a plateau ≥50 ng/mL (Fig. 1b). Based on this finding, to ensure maximal phosphorylation, we carried out subsequent stimulation using PDGF-BB at a concentration of 80 ng/mL. The largest fraction of phosphorylated receptor was seen following one minute of stimulation, the earliest time point measured (Fig. 1c). As exposure time to ligand was lengthened, the amount of phosphorylated receptor and total receptor decreased. In order to ensure reproducibility during subsequent stimulation, we chose a stimulation duration of 5 min.

### PDGFRα stimulation and downstream signaling is inhibited by nilotinib

**Fig. 2:**
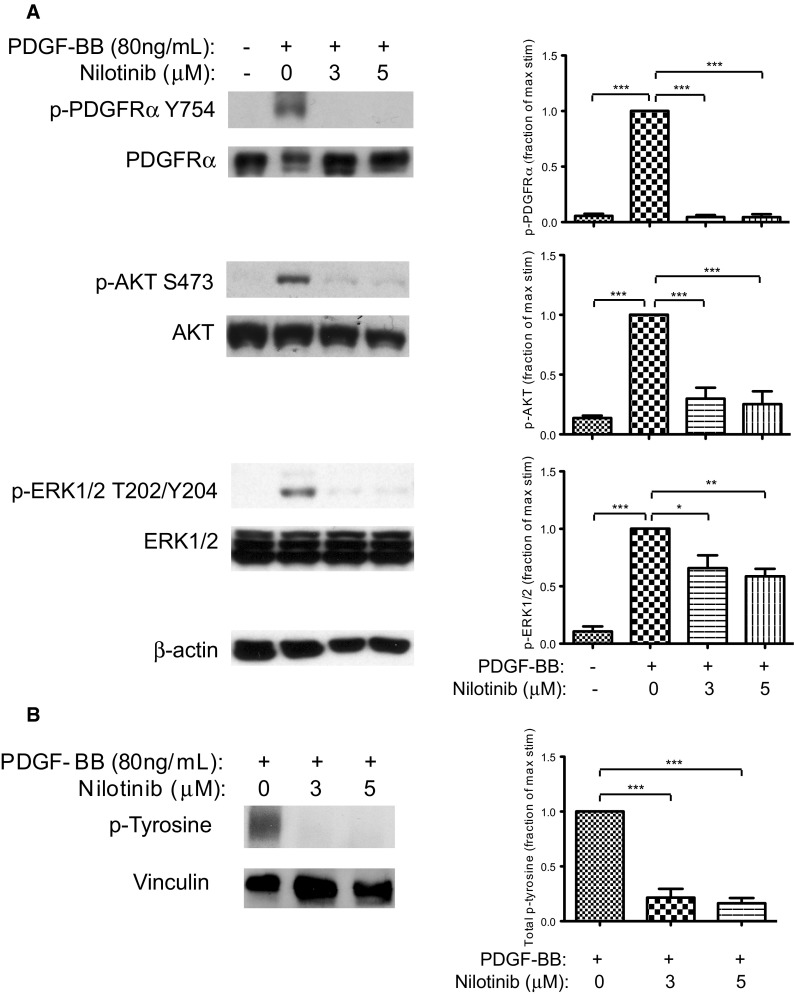
Nilotinib inhibits PDGF-BB-dependent activation of PDGFRα. SJ-G2 cultures in low-serum growth conditions were stimulated with exogenous PDGF-BB in the absence and presence of nilotinib. Accompanying bar graphs display relative phosphorylation in each condition, compared with stimulation in 0 μM nilotinib treatment (maximal stimulation). Significant difference is indicated by * for *p* < 0.05, ** for *p* < 0.01, and *** for *p* < 0.001. **a** Western blot analysis of PDGFRα, AKT and ERK1/2 phosphorylation. β-actin is loading control. Minimal basal phosphorylation of PDGFRα was observed, and little activation of downstream signaling mediators. Exposure to PDGF-BB resulted in phosphorylation of PDGFRα and activation of AKT and ERK1/2. Treatment with nilotinib decreased PDGFRα phosphorylation and the associated activation of downstream signaling mediators. **b** Immunoblotting of total cell lysate detected global reduction of phospho-tyrosine at the molecular weight of PDGFRα in the presence of nilotinib. Vinculin is loading control

We observed a dose-dependent effect on number of viable cells in culture between 0 and 3 μM nilotinib (see below), so chose an overlapping concentration range to examine signaling effects. To evaluate the effect of PDGFR activation on the downstream AKT and Ras pathways, we monitored phosphorylation of AKT S473 and ERK1/2 T202/Y204 in the presence and absence of nilotinib. In low-serum growth conditions, background phosphorylation of PDGFRα was minimal, and activation of AKT and ERK1/2 was not detected (Fig. 2a). Exposure to exogenous PDGF-BB resulted in increased phosphorylation of PDGFRα, as well as AKT and ERK1/2. The presence of 3 or 5 μM nilotinib during PDGF-BB stimulation resulted in little PDGFRα activation, and the phosphorylation of AKT and ERK1/2 were also significantly reduced. The activation of PDGFRα was evaluated using an antibody specific to phospho-tyrosine 754, but immunoblotting with a pan-phosphotyrosine antibody also demonstrated that phosphorylation of tyrosine residues at the molecular weight of PDGFR was generally reduced in the presence of nilotinib (Fig. 2b).

### Nilotinib inhibition of PDGFRα in serum decreases activation of AKT and ERK1/2 signaling

**Fig. 3:**
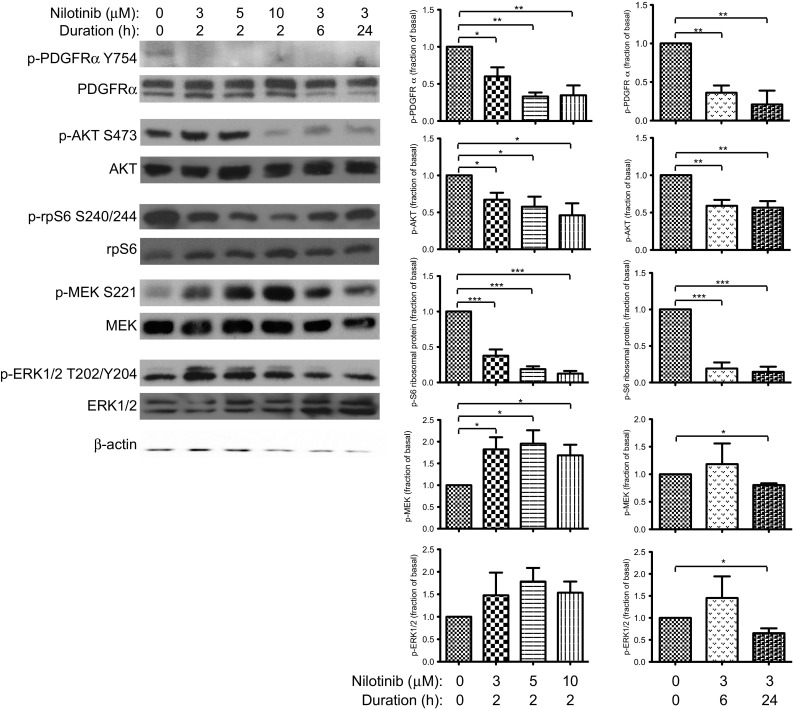
Nilotinib inhibits PDGFRα and the AKT and ERK1/2 signaling pathways. SJ-G2 cultures in 10 % FBS-supplemented growth media were exposed to nilotinib 0–10 μM for 2 h, and to 3 μM for up to 24 h. Phosphorylation of PDGFRα, AKT, S6 ribosomal protein, MEK and ERK1/2 was analyzed by Western blotting. β-actin is loading control. Accompanying bar graphs display relative phosphorylation in each condition, compared with basal levels (0 μM nilotinib vehicle treatment). Significant difference is indicated by * for *p* < 0.05, ** for *p* < 0.01, and *** for *p* < 0.001. Basal phosphorylation of all proteins was demonstrated. Exposure to nilotinib resulted in reduction of PDGFRα phosphorylation. Phosphorylation of AKT and S6 ribosomal protein was also decreased, and continually suppressed with extended inhibitor treatment. Phosphorylation of MEK was initially increased, but sustained exposure to nilotinib resulted in suppression below baseline. ERK1/2 activation was decreased following 24 h of nilotinib treatment

We then evaluated the effects of niloinib inhibition in the more complex milieu of serum-supplemented growth media, and additionally examined the phosphorylation of S6 ribosomal protein, which effects enhanced mRNA translation in AKT-stimulated cell growth, and of MEK, a direct activator of ERK1/2. In serum-supplemented growth media, a low basal level of PDGFRα phosphorylation was observed (Fig. 3, row 1 of graphs). Basal activation of AKT, S6 ribosomal protein, MEK and ERK1/2 was observed. To evaluate the effect of nilotinib on activation of PDGFRα and its signaling pathways, each inhibition condition was compared to this basal phosphorylation. Cultures were first exposed to 0–10 μM nilotinib for 2 h (Fig. 3, first column of graphs). With increasing nilotinib concentration, phosphorylation of PDGFRα was significantly decreased. During this time period, a small decrease in AKT activation was noted, as well as a marked decrease in S6 ribosomal protein phosphorylation. (Fig. 3, rows 2 and 3 of graphs). When exposure to 3 μM nilotinib was then extended to 6 and 24 h (Fig. 3, second column of graphs), a further reduction in AKT activation was seen, and the decreased phosphorylation of S6 ribosomal protein was sustained. Initial exposure to nilotinib resulted in stimulation of MEK activation (Fig. 3, row 4 of graphs). As exposure to 3 μM nilotinib was prolonged, however, MEK phosphorylation decreased, and after 24 h, was suppressed below baseline. ERK1/2 activation was not significantly altered during short-duration nilotinib treatment, but decreased following 24 h of exposure (Fig. 3, row 5 of graphs).

### Nilotinib decreases SJ-G2 proliferation

**Fig. 4:**
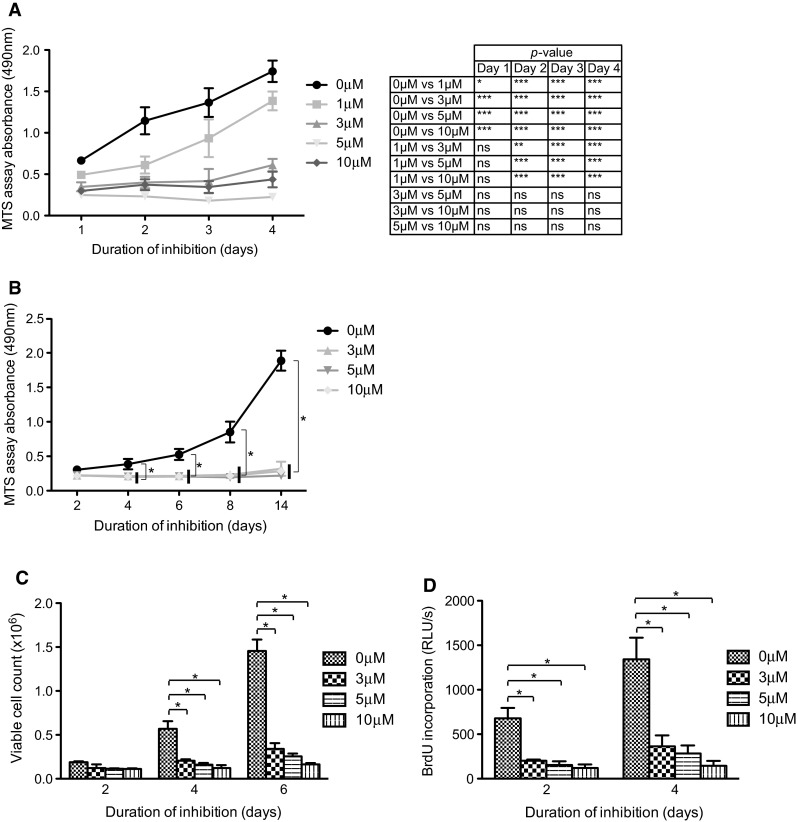
Nilotinib reduces SJ-G2 growth and proliferation. Cell cultures treated with nilotinib were compared with vehicle-treated controls. Significant difference is indicated by * for *p* < 0.05, ** for *p* < 0.01, and *** for *p* < 0.001. **a** SJ-G2 cultures were exposed to nilotinib (0–10 μM) for 1–4 days. MTS assay demonstrated a dose-dependent effect on numbers of viable cells in culture. All treated cultures demonstrated significantly fewer viable cells compared to control, and a further difference was seen between 1 μM and ≥3 μM nilotinib treatment. **b** Durability of effect on cell numbers was evaluated by exposing SJ-G2 cultures to nilotinib (0–10 μM) for 2–14 days. Treated cultures exhibited fewer viable cells up to 14 days. **c** SJ-G2 cultures were exposed to nilotinib (0–10 μM) for 2–6 days. The number of viable cells, as determined by exclusion of trypan blue dye, was lower in the presence of nilotinib. **d** SJ-G2 cultures were exposed to nilotinib (0–10 μM) for 2–4 days. Incorporation of BrdU as detected by ELISA was decreased in treated cultures

A dose-dependent effect on cell viability was demonstrated by MTS assay when SJ-G2 cells were treated with nilotinib (0–10 μM) (Fig. 4a). Following ≥1 day of exposure to the inhibitor, the viability of all treated cultures was less than that of vehicle-treated control. After ≥2 days of exposure, fewer viable cells were seen in cultures exposed to ≥3 μM nilotinib compared to cultures in 1 μM nilotinib. There was no consistent difference in effect on viable cell numbers between 3 and 10 μM nilotinib. To determine whether the effect on viability was sustained, SJ-G2 was cultured in the presence of nilotinib (0–10 μM) up to 14 days (Fig. 4b). Number of viable cells in treated cultures was significantly less than in vehicle-treated control, initially noted after 4 days of inhibition and continued through 14 days of exposure. This result was corroborated using direct counting of viable cells using trypan blue dye exclusion; when treated for 4 or more days, all SJ-G2 cultures exposed to ≥3 μM nilotinib contained significantly fewer viable cells compared to vehicle-treated control (Fig. 4c). There was no consistent difference among the cultures treated with 3–10 μM nilotinib. Apoptosis assays were also performed but no significant differences were noted (data not shown).

To evaluate the effect of nilotinib on SJ-G2 proliferation, we assessed the incorporation of BrdU by chemiluminescent ELISA (Fig. 4d). Following 2 and 4 days of exposure, all treated cultures (3–10 μM) generated significantly less luminescent product than vehicle-treated control.

### Nilotinib decreases in vitro and in vivo tumorigenicity

**Fig. 5:**
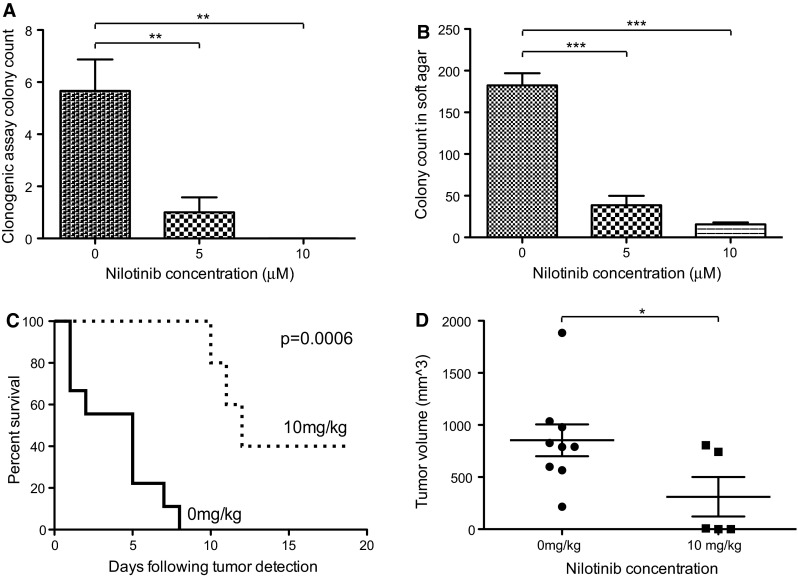
Nilotinib reduces SJ-G2 in vitro and in vivo growth. **a** Plate-attached SJ-G2 cells were exposed to nilotinib (0–10 μM) for 21 days. Colony numbers in the presence of nilotinib were compared to vehicle-treated controls; significant difference is indicated by ** for *p* < 0.01 and *** for *p* < 0.001. **b** SJ-G2 cells suspended in 0.35 % agar were exposed to nilotinib (0–10 μM) for 21 days. Colony numbers in the presence of nilotinib were compared to vehicle-treated controls; significant difference is indicated by *** for *p* < 0.001. **c** Kaplan–Meier plot for NOD–SCID mice bearing SJ-G2 flank tumors. Animals treated with nilotinib demonstrated prolonged survival. **d** Excised xenograft tumors that had been exposed to nilotinib were smaller than those from vehicle-treated mice. Significant difference is indicated by * for *p* < 0.05

The number of colonies formed in clonogenic assay was decreased in the presence of all concentrations of nilotinib (Fig. 5a). Anchorage-independent growth of SJ-G2 cells suspended in soft agar was also significantly decreased by exposure to nilotinib (Fig. 5b). Mice harboring SJ-G2 xenograft tumors survived significantly longer (*p* = 0.0006) when treated with nilotinib, compared with mice given drug vehicle (Fig. 5c). Tumors excised from treated mice were smaller at the time of animal sacrifice (Fig. 5d). Two treated animals did not become ill; at the end of the study period, the subcutaneous masses that had been identified were found to be fibrofatty tissue.

## Discussion

Demonstration of *PDGFRA* amplification as the most common CNA among pediatric HGG, and the association of histone H3.3 mutation in malignant pediatric brain-stem glioma with *PDGFRA* amplification, identified PDGFRα and its signaling pathways as likely integral to the malignant behavior of some tumors. It is therefore identified as a potential therapeutic target. Recognizing that understanding of this disease and advancement of therapy has historically been hindered by reliance upon studies of adult gliomas, we have carried out investigations of PDGFRα signaling and inhibition in cell lines derived from pediatric glioblastoma tumors.

Pediatric high-grade tumors exhibit variability in *PDGFRA* copy number, as well as in expression of PDGFRα and in platelet-derived growth factor (PDGF) itself [11, 29–31]. Although the SJ-G2 cell line does not contain amplification of *PDGFRA*, its expression of high levels of PDGFRα, the activation of which is dependent on dosage of ligand (PDGF-BB), suggests that it represents tumors that use PDGFRα signaling to support their tumorigenic behavior. To our knowledge, this is the first demonstration in a pediatric GBM cell line that activation of PDGFR is dependent upon ligand binding. This stands in contrast, for instance, to constitutively-activated mutant forms of epidermal growth factor receptor that are frequently found in adult GBM [32, 33]. Activation of PDGFRα occurred rapidly, with peak phosphorylation seen within a minute of exposure to PDGF-BB, and decayed, suggesting that normal mechanisms of receptor internalization and degradation remain functional in these cells [34].

Ligand-mediated PDGFR stimulation activates the PI3K–AKT and Raf–MEK–ERK signaling pathways [35–37] and is dependent upon cell type and developmental stage [38–40]. Stimulation with PDGF-BB of SJ-G2 cells resulted in phosphorylation of PDGFRα and corresponding activation of AKT and ERK1/2. Exposure to nilotinib inhibited PDGF-BB-mediated stimulation of the receptor, and markedly decreased activation of AKT and ERK1/2. Phosphorylation of tyrosine in response to PDGF-BB was generally decreased in the presence of nilotinib, suggesting that PDGFRα auto-activation was inhibited.

The effect of nilotinib inhibition was subsequently examined in the heterogeneous environment of serum-supplemented growth media. In this setting, we observed a low basal level of PDGFRα phosphorylation. Consistent with ongoing PDGFRα stimulation, we also detected basal activation of AKT, S6 ribosomal protein, MEK and ERK1/2, although activation of other cell-surface receptors may have contributed to this. Exposure to nilotinib decreased PDGFRα phosphorylation in all conditions. Phosphorylation of AKT and S6 ribosomal protein was rapidly reduced, and was maintained at a significantly decreased level during sustained inhibitor exposure, suggesting that some component of their activation is PDGFR-dependent. Interestingly, there was an early increase in MEK phosphorylation, within the first 2 h of nilotinib exposure. The rapidity of this change suggests that it results from an alteration in steady-state balance between signaling pathways.

Since inhibition with nilotinib suppressed PDGFRα-mediated signaling, we then investigated its effect on SJ-G2 cell numbers and proliferation. Our results show that cell proliferation and clonogenic potential (in vitro tumor-forming ability) were significantly reduced at 3 μM nilotinib compared to vehicle-treated control. The reduced proliferation resulted in fewer viable cells over the same time interval of growth compared to untreated controls. As patients receiving typical oral dosaging of nilotinib achieve a mean peak serum concentration of 3.6 μM [24], the plateau in effect suggests that maximal anti-PDGFRα effect occurs at a concentration that can be achieved safely in the clinical context. The in vitro results were supported by in vivo findings of prolonged survival in nilotinib-treated mice bearing SJ-G2 tumors, and smaller tumor sizes in the treated mice.

These functional assays indicate that the result of nilotinib-mediated decrease in PDGFRα activation and signaling is a reduction in SJ-G2 proliferation, colony formation and in vivo growth. This overall decrease in tumorigenic behavior suggests that SJ-G2 may represent tumors that depend upon PDGFRα signaling and would therefore respond to nilotinib inhibition.

In contrast, SF-188 expressed very little PDGFRα and demonstrated high basal activation of AKT and ERK1/2, suggesting that this cell line is dependent upon activation of alternative growth factor receptor pathways to transduce mitogenic signals (Supplementary Figs. 2S, 3S, 4S). Consistent with this, we found that treatment with nilotinib had little effect on activation of AKT and ERK1/2. Furthermore, while treated cultures of SF-188 initially demonstrated lower viability, this difference was lost with sustained inhibition. SF-188 contains a mutation of the tumor suppressor and Ras-GAP NF1 [41], which likely maintains activation of both AKT and ERK1/2 pathways by failing to regulate Ras activation. In contrast to SJ-G2, which contains wildtype NF1, SF-188 can be seen to represent the subset of tumors that do not depend upon PDGFRα activation.

All chemotherapeutic agents intended for use in the CNS must be able to penetrate the blood–brain barrier. Although its predecessor imatinib has poor CSF penetration [42], regarding this question nilotinib remains under clinical investigation. Interestingly, nilotinib demonstrates in vivo activity against Abelson tyrosine kinase (Abl) within neurons of murine basal ganglia [23].

As our understanding of the biology of pediatric HGG evolves, so does our recognition that they are a clinical and pathological entity distinct from adult tumors—and, indeed, that they encompass different subtypes. In this study, we identified the SJ-G2 cell line as representative of tumors that are dependent upon PDGFRα expression and activation to drive signaling pathways leading to tumorigenic behavior, and which are therefore most likely to be responsive to inhibition with nilotinib. Based on these data, we suggest that a subset of appropriately-identified pediatric HGG should be considered candidates for nilotinib treatment.

## Electronic supplementary material

Below is the link to the electronic supplementary material.

Supplementary material 1 (TIFF 18722 kb)

Supplementary material 2 (TIFF 46470 kb)

Supplementary material 3 (TIFF 48791 kb)

Supplementary material 4 (TIFF 83603 kb)

Supplementary material 5 (DOC 55 kb)
