# Supplementary figures and images for: A preclinical study demonstrating the efficacy of nilotinib in inhibiting the growth of pediatric high-grade glioma
Source: J Neurooncol. 2015 Mar 4;122(3):471–80. doi: 10.1007/s11060-015-1744-y (PMC4436849; doi:10.1007/s11060-015-1744-y)

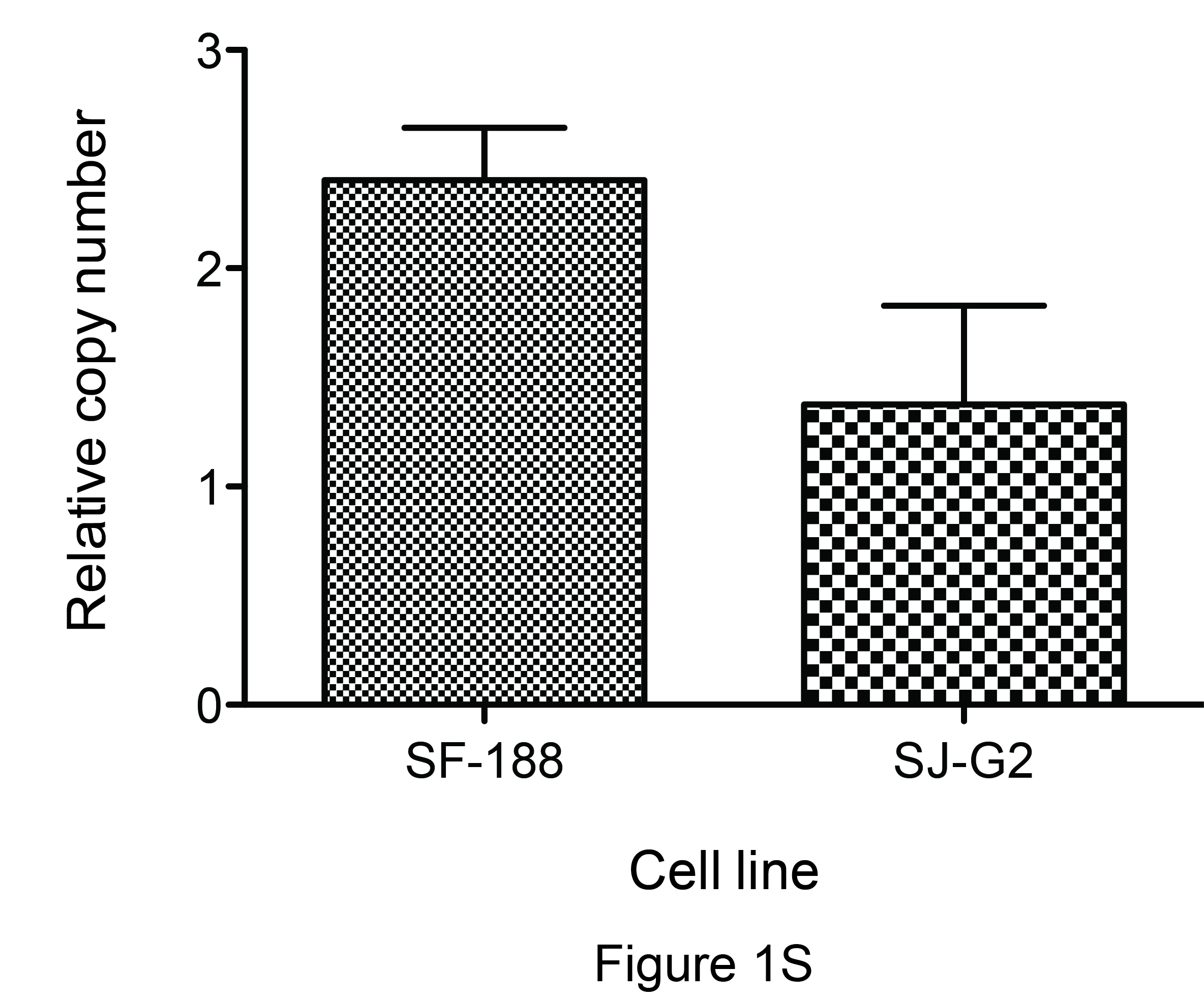

Supplement: Supplementary file 1 — Supplementary material 1 (TIFF 18722 kb) [file 11060_2015_1744_MOESM1_ESM.tif]

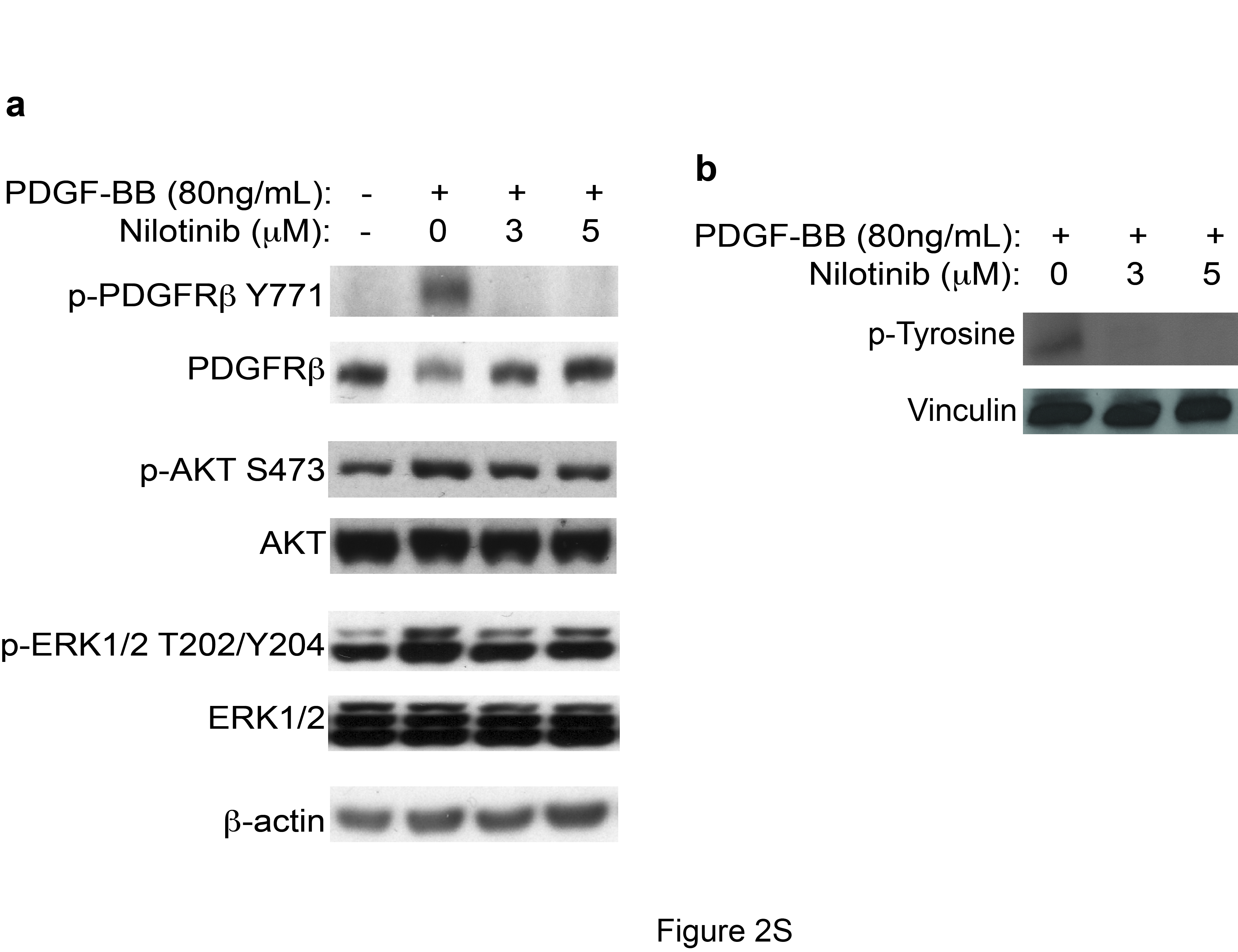

Supplement: Supplementary file 2 — Supplementary material 2 (TIFF 46470 kb) [file 11060_2015_1744_MOESM2_ESM.tif]

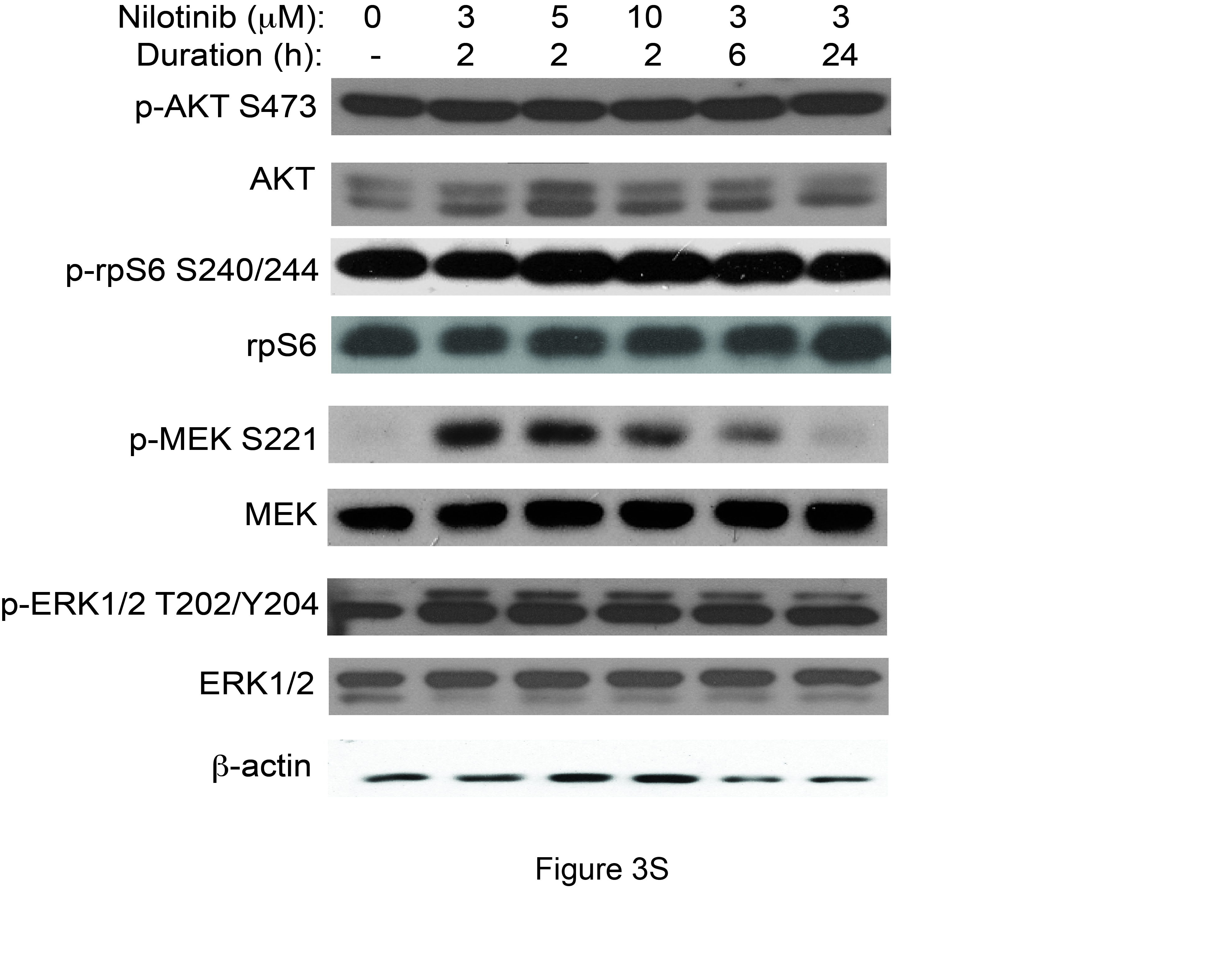

Supplement: Supplementary file 3 — Supplementary material 3 (TIFF 48791 kb) [file 11060_2015_1744_MOESM3_ESM.tif]
