# Supplementary material for: A preclinical study demonstrating the efficacy of nilotinib in inhibiting the growth of pediatric high-grade glioma
Source: J Neurooncol. 2015 Mar 4;122(3):471–80. doi: 10.1007/s11060-015-1744-y (PMC4436849; doi:10.1007/s11060-015-1744-y)
Supplement: Supplementary file 5 — Supplementary material 5 (DOC 55 kb) [file 11060_2015_1744_MOESM5_ESM.doc]

**A Preclinical Study Demonstrating the Efficacy of Nilotinib in Inhibiting the Growth of Pediatric High-Grade Glioma**

Karolyn Au^1^, Sanjay K. Singh^1^, Kelly Burrell^1^, Nesrin Sabha^1^, Cynthia Hawkins^1^, Annie Huang^1^ and Gelareh Zadeh^1^*

1 The Arthur and Sonia Labatt Brain Tumor Research Centre, Hospital for Sick Children, University of Toronto, Toronto, ON M5G 1L7, Canada.

***Corresponding Author**

Dr. Gelareh Zadeh

Division of Neurosurgery, Toronto Western Hospital,

4-439 West Wing, TWH399 Bathurst Street, Toronto, ON, M5T2S8

Phone: +1-416-6035679, Fax: +1-416-6035298 E-Mail: [gelareh.zadeh@uhn.ca](mailto:gelareh.zadeh@uhn.ca).

**Supplementary methods**

**Evaluation of exogenous ligand stimulation and inhibition**

Response to ligand concentration was determined by incubating cultures for 5 minutes with minimal media containing 0, 10, 20, 50, 80 or 100ng/mL PDGF-BB. Response to duration of stimulation was determined by incubating cultures with minimal media containing 80ng/mL PDGF-BB for 0, 1, 5, 10, 30 or 60 minutes. Subsequent stimulation experiments were carried out using PDGF-BB 80ng/mL for 5 minutes.

For evaluation of inhibitor effect on cell stimulation, cultures were incubated with nilotinib (0, 3 or 5μM; provided by Novartis Pharma AG, Basel) in low-serum media for 2 hours prior to ligand stimulation. Cultures were then exposed to minimal media containing PDGF-BB and nilotinib, as described above. For all other analyses, cell cultures in regular growth media were exposed to nilotinib (0-10μM) for the indicated times. All stimulation and inhibition experiments were carried out in at least three independent replicates.

**Western immunoblot assay**

Cell cultures were scraped and lysed on ice in 1X Cell Lysis Buffer (Cell Signaling) containing protease (Roche Diagnostics) and phosphatase inhibitors (Calbiochem). Protein was quantified using bicinchoninic acid (BCA) assay (Thermo Scientific), and lysate containing 25-40μg protein was loaded onto 7-12.5% SDS-PAGE gels for electrophoresis. Proteins were transferred to PVDF membrane (PerkinElmer) using a semi-dry transfer apparatus (Bio-Rad Laboratories). Membranes were blocked in 5% non-fat milk, and incubated overnight at 4°C with primary antibodies in 3% bovine serum albumin (BSA) unless otherwise indicated. Antibodies are as follows: β-actin (1:25000; #A2228, Sigma-Aldrich), AKT (1:1000; #9272, Cell Signaling), p-AKT S473 (1:1000; #4058, Cell Signaling), ERK1/2 (1:1000; #9102, Cell Signaling), p-ERK1/2 T202/Y204 (1:1000 in 5% non-fat milk; #9106, Cell Signaling), MEK1/2 (1:1000; #9126, Cell Signaling), p-MEK1/2 S221 (1:2000; #2338, Cell Signaling), PDGFRα (1:500; #3174, Cell Signaling), p-PDGFRα Y754 (1:2000; #5460, Abcam), PDGFRβ (1:500; #3169, Cell Signaling), p-PDGFRβ Y771 (1:500; #3173, Cell Signaling), S6 ribosomal protein (1:1000; #2317, Cell Signaling), p-S6 ribosomal protein S240/S244 (1:1000; #2215, Cell Signaling), and vinculin (1:500; SAB4503069, Sigma-Aldrich). Membranes were washed with tris-buffered saline (TBS) containing 0.1% Tween-20, incubated with horseradish peroxidase-conjugated secondary antibody (Bio-Rad) specific to each primary antibody, and detected using Western Lightning® Plus-ECL (PerkinElmer). Detection of bands was carried out using the FluorChemQ MultiImage III System (AlphaInnotech), and chemiluminescent signal quantification was performed using AlphaView® Software v.3.0.0.0 (AlphaInnotech). Automatic exposure time was determined by the software based on light and filter settings, and luminescence quantification of freshly-developed membranes performed in the linear range. Multiplex band analysis and uniform background subtraction were performed manually. Phosphorylation level was defined as the ratio of band intensity of phosphorylated protein to total protein on the same membrane (stripped and re-incubated with fresh antibody). Each experimental condition was compared to a control condition as described. Analysis was performed by one-way ANOVA followed by Dunnett’s *post hoc* test to compare each experimental condition to control condition. Figures show a representative Western blot, alongside graphs displaying mean ± SEM for minimum three replicate experiments. Significance is denoted by * for p<0.05, ** for p<0.01, and *** for p<0.001.

**Viability assays**

To evaluate the dose effect of nilotinib on cell viability, SJ-G2 cells (3x10^3^) were seeded in 96-well plates and allowed to equilibrate overnight. Cultures were exposed to nilotinib (0-10μM) for 1-4 days, then mixed with CellTiter 96® Aqueous One Solution reagent (Promega) according to the manufacturer’s instructions; this assay uses the reduction of the tetrazolium compound 3-(4,5-dimethylthiazol-2-yl)-5-(3-carboxymethoxyphenyl)-2-(4-sulfophenyl)-2H-tetrazolium (MTS) to a colored formazan product as a marker of cell viability. Following 4 hours of incubation, absorbance at 490nm was read using a VersaMax tunable plate reader (Molecular Devices). To evaluate the effect of sustained nilotinib exposure on cell viability, SJ-G2 (2x10^3^) cells were seeded in 96-well plates and allowed to equilibrate overnight. Cultures were exposed to nilotinib (0-10μM) for 2-14 days, then mixed with CellTiter 96® Aqueous One Solution reagent (Promega). Following 4 hours of incubation, absorbance at 490nm was read using the VersaMax plate reader. Each condition was replicated in six wells, in at least three independent experiments.

To directly evaluate viable cell numbers using trypan blue dye exclusion, SJ-G2 cells (1x10^5^) were seeded in 6-well plates and allowed to equilibrate overnight. Cultures were exposed to nilotinib (0-10μM) for 2, 4 or 6 days. All cells in each well were collected, and evaluated for trypan blue exclusion using the Vi-CELL Cell Viability Analyzer (Beckman Coulter). Each condition was evaluated in triplicate, in at least three independent experiments.

**Cell proliferation assay**

SJ-G2 cells (2x10^3^) were seeded in 96-well plates and allowed to equilibrate overnight. Cultures were exposed to nilotinib (0-10μM) for 2 or 4 days. Chemiluminescent Cell Proliferation ELISA (Roche) using 5-bromo-2’-deoxyuridine (BrdU) labeling was carried out according to the manufacturer’s instructions, using a 12-hour labeling period. Chemiluminescent signal was read using SpectraMax Gemini EM plate reader (Molecular Devices) in relative luminescent units per second (RLU/s). Each condition was replicated in six wells, in at least three independent experiments.

**Colony-formation assays**

The effect of nilotinib on *in vitro* growth potential was evaluated using clonogenic assay. SJ-G2 cells (250) were seeded in 6-well plates and grown in the presence of nilotinib (0-10μM) for 3 weeks, with nilotinib-containing media refreshed every 3 days. Colonies were fixed with 10% formalin and stained with 0.05% crystal violet. Anchorage-independent growth was evaluated using soft-agar colony-forming assay as previously described [[23]](#_ENREF_23). SJ-G2 cells (2.5x10^4^) were suspended in RPMI containing 0.35% agar, 10% FBS and antibiotic-antimycotic (Wisent), and applied to a base agar layer. Growth media containing nilotinib (0-10μM) was applied to the agar surface. Plates were incubated for 21 days, and nilotinib-containing media refreshed every 3 days. Colonies in agar were fixed in 10% formalin, stained with 0.05% crystal violet, and those >50μm in size were counted. Each condition was carried out in triplicate, in three independent experiments.

**Statistical analysis**

All experiments were performed minimum in triplicate with mean and standard error of the mean reported where appropriate. One- or two-way analysis of variance (ANOVA) was conducted for multi-group comparisons, followed by Dunnett’s (groups compared to one control group) or Tukey’s (to identify differences among sub-groups) *post hoc* test. Where appropriate, direct comparisons were conducted using unpaired two-tailed t-test. Significance was defined by * for *p*<0.05, ** for *p*<0.01, and *** for *p*<0.001.

**PDGFRA copy number evaluation**

Genomic DNA was isolated from SJ-G2, SF-188 and NHA using the DNeasy Blood & Tissue Kit (Qiagen), and quantified on the Nanodrop ND-1000 Spectrophotometer (Thermo Scientific). Quantitative polymerase chain reaction (qPCR) analysis was carried out on a StepOnePlus Real-Time PCR System (Applied Biosystems) using the Platinum SYBR Green qPCR Supermix-UDG (Invitrogen). Primer efficiency was determined by standard curve. Copy number was determined using the Pfaffl method, and copy number compared to NHA. The endogenous control was *GPX7*, which is seen at normal copy number in pHGG tumor sample [26]. Primers used were as follows: *PDGFRA* forward 5’-TGGTACCTGCCTCCTACGAC-3’ and reverse 5’-CACGCACCTTATGATTTTGC-3’, *GPX7* forward 5’-ATTCTGGTTGGAGTGGTCTG-3’ and 5’-AAAAGGAAGGAGAGCAAAGC-3’*.*

**Evaluation of stimulation, activation and inhibition in SF-188**

Stimulation with exogenous PDGF-BB and nilotinib inhibition was carried out in SF-188 as described in Methods and Materials for SJ-G2, with changes described as follows. For evaluation of nilotinib dose effect by MTS assay, 1x10^3^ SF-188 cells were seeded in 96-well plates. To evaluate the effect of sustained nilotinib exposure, 5x10^2^ SF-188 cells were seeded in 96-well plates. Proliferation assay by BrdU incorporation was carried out using 5x10^2^ SF-188 cells seeded in 96-well plates. For clonogenic assay, SF-188 cultures were incubated for 1 week prior to fixation.

**Supplementary results**

**PDGFRA is not amplified in SJ-G2 and SF-188**

Copy number of *PDGFRA* was determined in SJ-G2 and SF-188 using qPCR and compared to NHA. Although copy number was increased two-fold in SF-188, and despite the increased expression of PDGFRα in SJ-G2, neither cell line was seen to harbor high-level amplification of the gene (Supp. Fig. 1S).

**AKT and ERK1/2 activation in SF-188 is independent of PDGFR**

In low-serum growth conditions, minimal phosphorylation of PDGFRβ was observed (Supp. Fig. 2SA). Basal activation of AKT and ERK1/2 was seen. Stimulation with exogenous PDGF-BB resulted in phosphorylation of PDGFRβ, but only slight increase in activation of AKT and ERK1/2. Treatment with nilotinib resulted in minimal PDGF-BB stimulation of PDGFRβ phosphorylation. Although the PDGF-BB-mediated increase in AKT and ERK1/2 phosphorylation was attenuated by nilotinib, these signaling mediators remained strongly phosphorylated. Probing with pan-phospho-tyrosine antibody suggested that PDGFR tyrosine phosphorylation was globally reduced by nilotinib treatment (Supp. Fig. 2SB).

**Nilotinib does not inhibit AKT and ERK1/2 activation in SF-188**

In serum-supplemented growth conditions, basal PDGFR phosphorylation was not detected in SF-188 (data not shown). Nevertheless, high levels of activated AKT, S6 ribosomal protein and ERK1/2 were observed (Supp. Fig. 3S). Exposure to nilotinib even at 10μM did not alter the phosphorylation of these signaling mediators, and continuous incubation with 3μM nilotinib for 24 hours also did not alter their activation. MEK activation was low at baseline, and nilotinib treatment initially resulted in an increase in activation. With inhibitor exposure to 24 hours, however, this activation was reduced to baseline.

**Nilotinib reduces viability and proliferation of SF-188**

In SF-188 exposed to nilotinib for 2-14 days, lower activity on MTS assay was observed in treated cultures between 4 and 8 days (Supp. Fig. 4SA). At 14 days, however, no significant difference was seen between nilotinib-exposed and vehicle-treated cultures at 14 days. When assessed by trypan blue exclusion, we observed a decrease in viable cell numbers only in cultures treated with high-dose (10μM) nilotinib (Supp. Fig. 4SB). A reduction in proliferation was seen in all cultures treated with nilotinib (3-10μM) when evaluated by BrdU incorporation (Supp. Fig. 4SC).

**Supplementary figure legends**

**Supplementary Fig. 1** SJ-G2 and SF-188 do not contain amplification of *PDGFRA*. Real-time PCR determination of *PDGFRA* copy number in pHGG cell lines SJ-G2 and SF-188, as compared to NHA, does not demonstrate high-level amplification of the gene.

**Supplementary Fig. 2** AKT and ERK1/2 activation is PDGFR-independent in SF-188. SF-188 cultures in low-serum growth conditions were stimulated with exogenous PDGF-BB in the absence and presence of nilotinib. (a) Western blot analysis of phospho/total PDGFRβ, AKT and ERK1/2, and β-actin as loading control. Minimal phosphorylation of PDGFRβ was observed in low-serum growth conditions, but high basal levels of activated AKT and ERK1/2 were detected. Exposure to exogenous PDGF-BB resulted in phosphorylation of PDGFRβ, and small increases in activation of AKT and ERK1/2. Nilotinib inhibited PDGF-BB stimulation of PDGFR, but had little effect on activation of signaling mediators. (b) Immunoblotting of cell lysate demonstrated reduction of phospho-tyrosine at the molecular weight of PDGFR in the presence of nilotinib. Vinculin was used as loading control.

**Supplementary Fig. 3** Nilotinib does not inhibit AKT and ERK1/2 activation in SF-188. SF-188 cultures in 10% FBS-supplemented media were exposed to nilotinib at the concentrations and for the intervals indicated. Basal activation of AKT and ERK1/2 signaling pathways persisted in the presence of nilotinib.

**Supplementary Fig. 4** SF-188 viable cell numbers and proliferation are decreased by nilotinib. (a) SF-188 cultures were exposed to nilotinib (0-10μM) for 2-14 days. MTS assay detected lower viability in nilotinib-treated cultures compared to vehicle-treated control. With sustained exposure, however, the difference was no longer observed. (b) SF-188 cells were exposed to nilotinib (0-10μM) for 2-6 days, and viability determined by exclusion of trypan blue dye. The number of viable cells was reduced only in the presence of the highest concentration of nilotinib, compared to vehicle-treated control. (c) SF-188 cells were exposed to nilotinib (0-10μM) for 2-4 days. Incorporation of BrdU as detected by ELISA was decreased in treated cultures compared to vehicle-treated control.
